# Supplementary figures and images for: Flexible Training Planning Coupled with Flexible Assessment: A 12-Week Randomized Feasibility Study in a Youth Female Volleyball Team
Source: Children (Basel). 2022 Dec 24;10(1):29. doi: 10.3390/children10010029 (PMC9856447; doi:10.3390/children10010029)

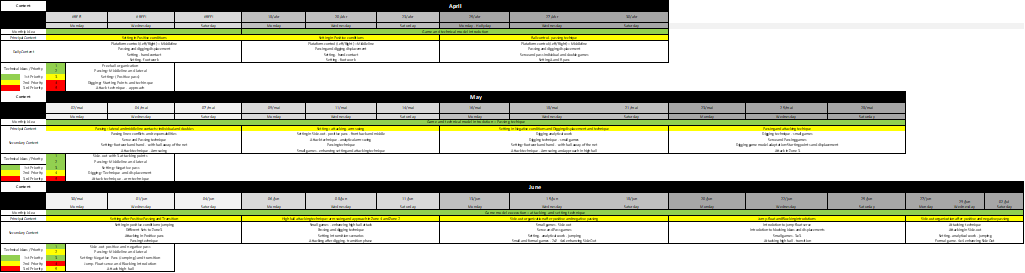

Supplement: Supplementary file 1 [file children-10-00029-s001.zip › Figure S5 Periodized Planning.tif]

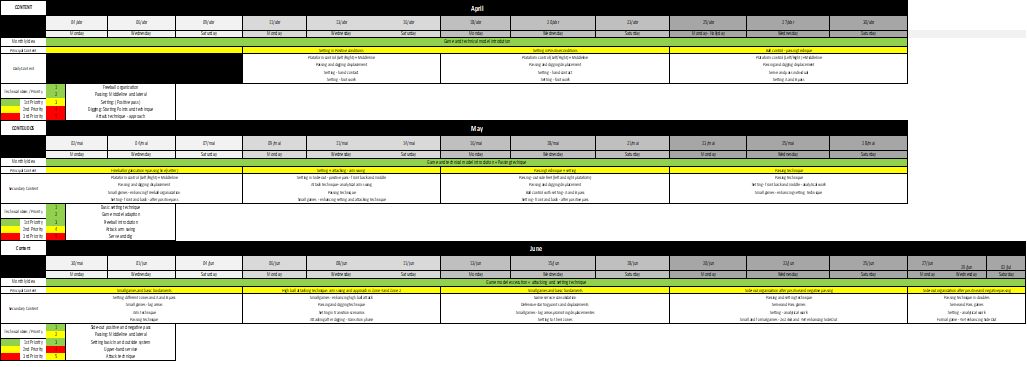

Supplement: Supplementary file 1 [file children-10-00029-s001.zip › Figure S6 Flexible Planning.tif]

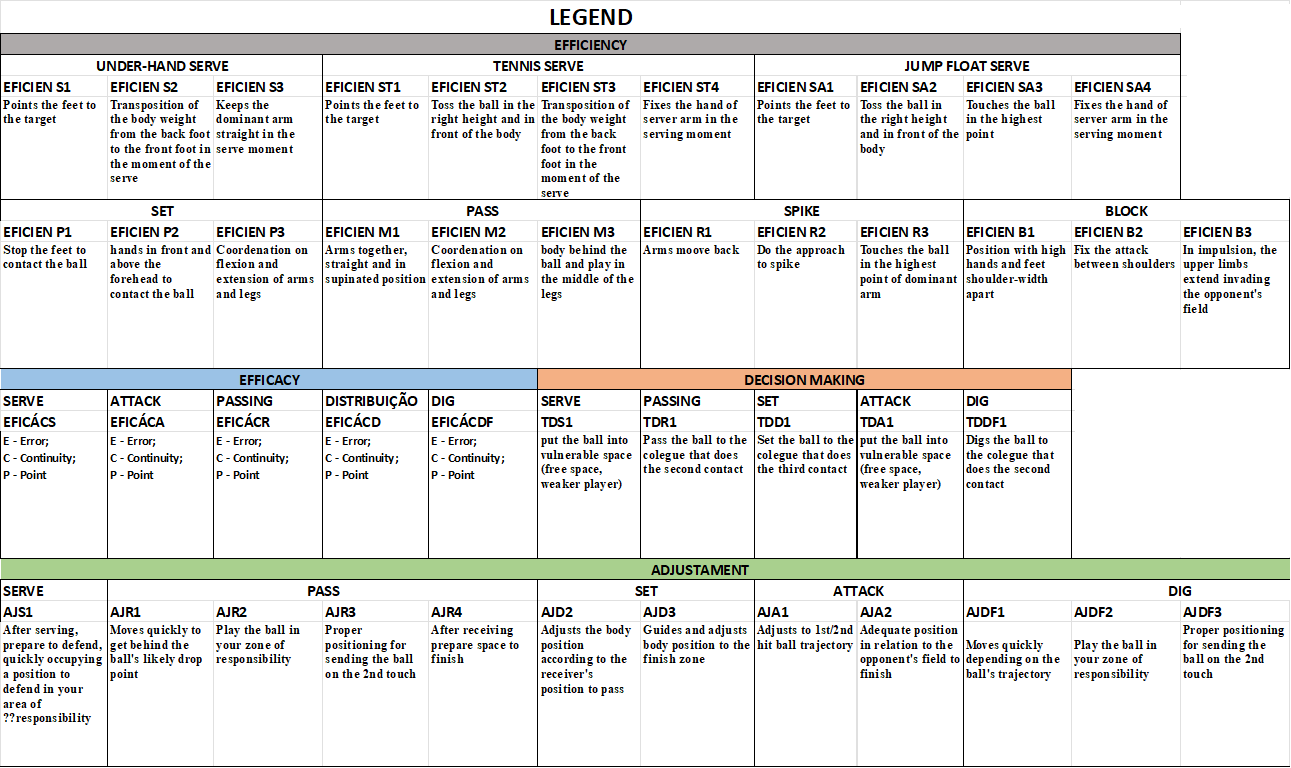

Supplement: Supplementary file 1 [file children-10-00029-s001.zip › Figure S7 GPAI I.tif]

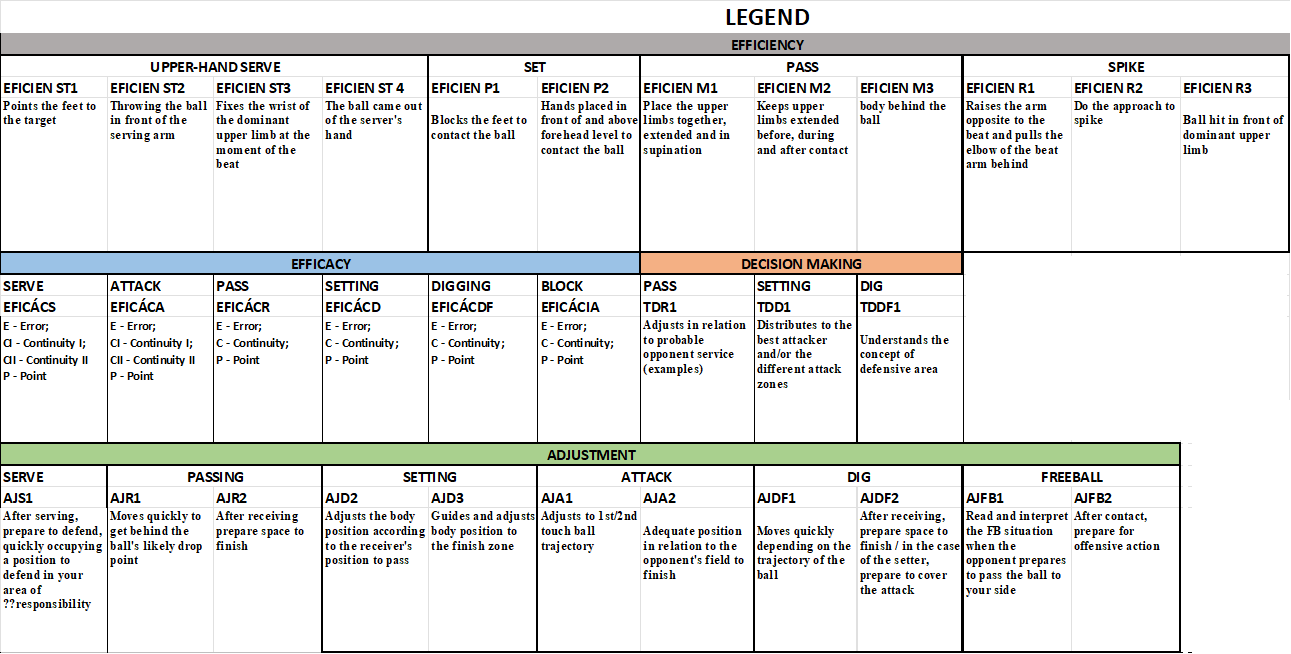

Supplement: Supplementary file 1 [file children-10-00029-s001.zip › Figure S8 GPAI II.tif]

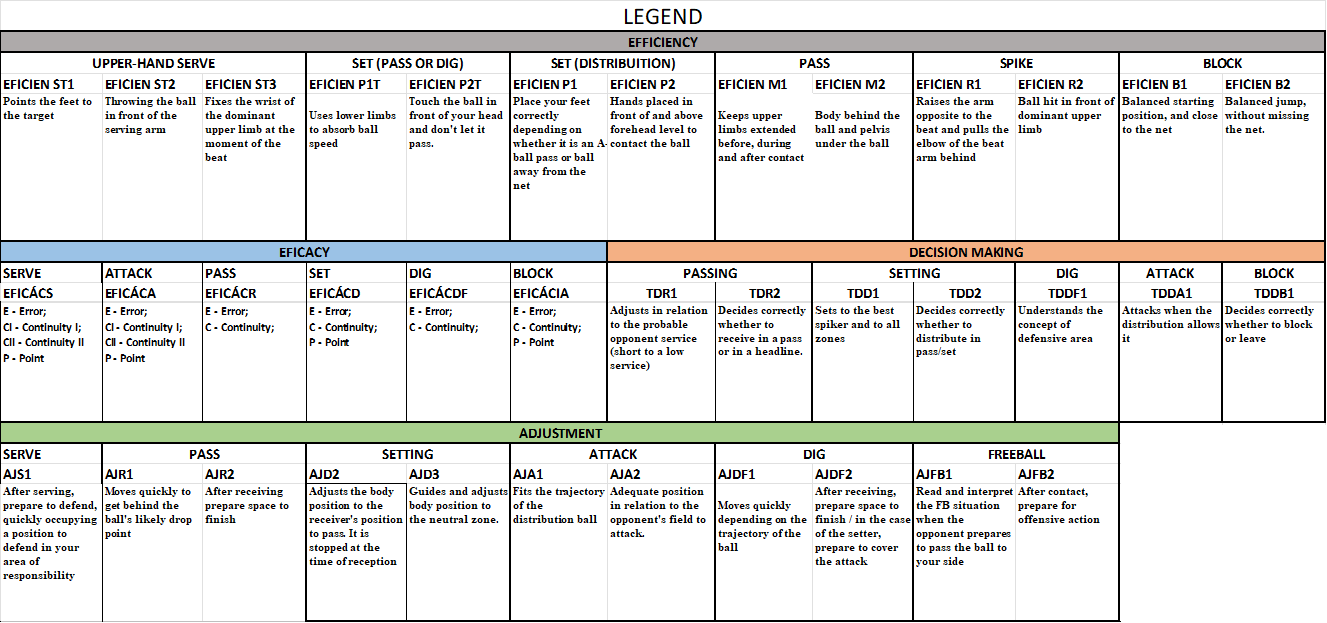

Supplement: Supplementary file 1 [file children-10-00029-s001.zip › Figure S9 GPAI III.tif]

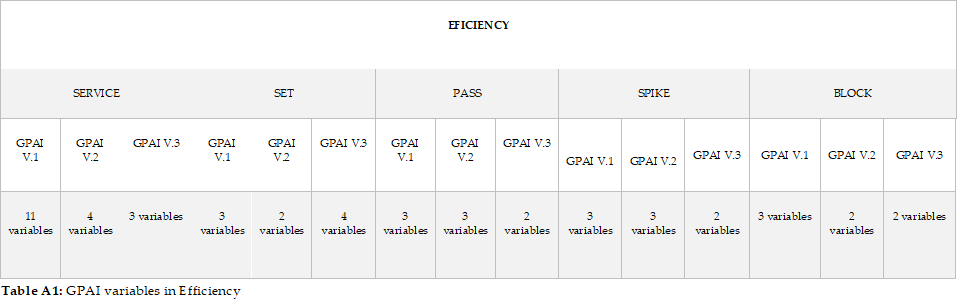

Supplement: Supplementary file 1 [file children-10-00029-s001.zip › Table S1.tif]

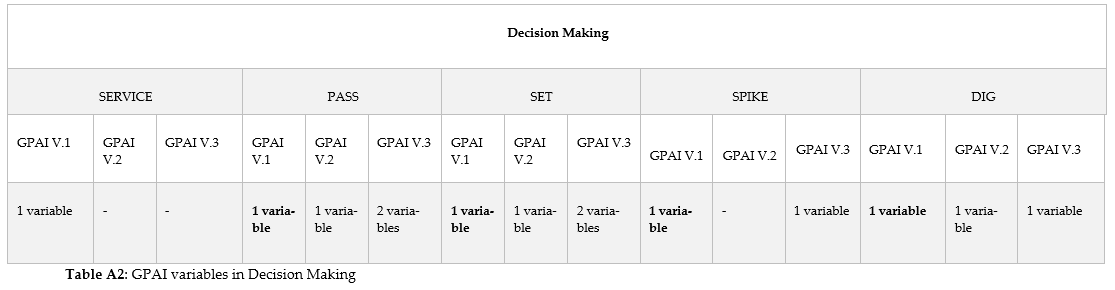

Supplement: Supplementary file 1 [file children-10-00029-s001.zip › Table S2.tif]
